# Neurological basis of poor insight in psychosis: A voxel-based MRI study

**DOI:** 10.1016/j.schres.2008.04.022

**Published:** 2008-08

**Authors:** Michael A. Cooke, Dominic Fannon, Elizabeth Kuipers, Emmanuelle Peters, Steven C. Williams, Veena Kumari

**Affiliations:** a Department of Psychology, Institute of Psychiatry, King's College London, London, UK; b Division of Psychological Medicine, Institute of Psychiatry, King's College London, London, UK; c NIHR Biomedical Research Centre for Mental Health, South London and Maudsley Foundation NHS Trust, London, United Kingdom; d Centre for Neuroimaging Sciences, Institute of Psychiatry, King's College London, London, UK

**Keywords:** Insight, Psychosis, Temporal lobe, Parietal lobe, Precuneus

## Abstract

**Background:**

As a reflection of poor insight, people with schizophrenia often disagree with carers and clinicians about whether (a) their experiences are abnormal, (b) they are mentally ill, and (c) they need treatment.

**Methods:**

This study used voxel-based morphometry to identify the associations between total and regional grey matter volumes and self-reported and observer-rated insight in 52 patients with schizophrenia or schizoaffective disorder. Thirty healthy participants were also studied.

**Results:**

There were positive associations in patients between (i) the ability to recognise experiences as abnormal and the total and right superior temporal gyrus grey matter volumes, (ii), awareness of problems (‘something wrong’) and the left precuneus grey matter volume and (iii) awareness of symptoms and attributing them to illness and grey matter volumes in the left superior–middle temporal gyrus and the right inferior temporal and lateral parietal gyri. The ‘recognition of the need for medication’ dimension did not correlate with total or any regional grey matter volumes. Relative to controls, patients had less total and regional grey matter volumes in the thalamus and middle occipital and superior temporal gyri.

**Conclusions:**

Lower grey matter volumes in the temporal and parietal regions that have been implicated in self-monitoring, working memory and access to internal mental states are associated with poor insight on certain dimensions in psychosis.

## 1 Introduction

People with schizophrenia often disagree with their carers and clinicians about whether (a) their experiences and behaviours are abnormal, (b), they are mentally ill and (c) they are in need of psychiatric treatment (David, 1990). Such disagreements are considered to reflect poor insight on the part of the patient. Apparent similarities between awareness deficits in neurological disorders and poor insight in schizophrenia have led to the suggestion that they have a comparable neurological basis (Amador et al., 1991, McGlynn and Schacter, 1997). Evidence for this suggestion has come primarily from studies showing significant relationships between measures of insight and performance on neuropsychological tests of general as well as specific cognitive domains (review, Cooke et al., 2005) which in turn are associated with the volume (review, Antonova et al., 2004) and functional response of specific brain regions (review, Shad et al., 2007).

The use of modern neuroimaging methods has allowed the neurobiological basis of insight in schizophrenia to be investigated more directly. Early neuroimaging studies of insight, which tended to use global brain measurements, found relationships between poor insight and ventricular enlargement (Takai et al., 1992) and smaller total brain volume (Flashman et al., 2000), although other studies did not replicate these relationships (David et al., 1995, Rossell et al., 2003).

Using more sophisticated methodologies, region of interest (ROI) studies have found a number of associations between frontal brain regions and insight. One study (Laroi et al., 2000) involving 21 patients with schizophrenia linked frontal cortical atrophy to poor insight assessed with the Scale to Assess Unawareness of Mental Disorders (SUMD, Amador et al., 1993). Another study (Flashman et al., 2001) examined the associations between insight, again assessed with the SUMD, and eight frontal lobe sub-regions [frontal pole, superior frontal gyrus, middle frontal gyrus, inferior frontal gyrus, orbital frontal gyrus, precentral gyrus, gyrus rectus and cingulate] in a group of 15 patients with schizophrenia; the findings revealed large negative associations between awareness of symptoms and bilateral middle frontal gyrus volumes, and between misattribution of symptoms and bilateral superior frontal gyrus volumes. Shad et al. (2004) reported smaller dorsolateral prefrontal cortex (DLPFC) volumes as well as poor performance on the Wisconsin Card Sorting Test in first-episode psychosis patients with poor insight (*n* = 18; assessed with a single question, derived from the insight item of the Hamilton Depression Rating Scale; Hamilton, 1960) relative to those with good insight (*n* = 17). A recent study by the same group involving 14 antipsychotic naïve first-episode schizophrenia patients, and using the SUMD to measure insight, found associations between smaller right DLPFC volumes and impaired awareness of symptoms but also between *larger* right medial orbitofrontal volumes and misattribution of symptoms (Shad et al., 2006). More recently, we (Sapara et al., 2007) observed associations between smaller total prefrontal grey matter volumes and lower insight into the presence of illness and symptoms assessed using the Birchwood Insight Scale (Birchwood et al., 1994), and the Schedule for the Assessment of Insight — Expanded (Kemp and David, 1997), in a sample of 28 chronic patients with schizophrenia.

A number of recent studies have employed voxel-based morphometry (VBM) (Ashburner and Friston, 2000, Good et al., 2001). For example, Ha and colleagues reported a negative association between insight and grey matter concentrations bilaterally in the (middle) cingulate and temporal regions in 35 paranoid schizophrenia patients (illness duration 0.1 to 15 years) (Ha et al., 2004). However, this study used a unidimensional measure of insight (the G12 item of the Positive and Negative Symptoms Scale, Kay et al., 1987), so it is not clear which aspects of insight may be related to grey matter volume in the temporal lobe. Bassitt and colleagues failed to find any relationship between insight assessed with the SUMD and regional brain volumes examined with VBM in a sample of 50 chronic patients with schizophrenia (Bassitt et al., 2007).

The present study was designed to examine the associations between regional grey matter volumes and dimensions of insight in schizophrenia assessed with a self-rated (Birchwood Insight Scale, Birchwood et al., 1994) and an observer-rated insight measure (the Schedule for the Assessment of Insight — Expanded, Kemp and David, 1997). A factor analysis was undertaken to combine these measures, and is described in detail elsewhere (Cooke et al., 2007). The four factors derived from this analysis were: Awareness of and Attribution to Illness, Recognition of the Need for Medication, Awareness of Problems, and Symptom Re-labelling (see Methods and materials for greater details). The focus of our study was on investigating associations between total and regional grey matter volumes and insight factors in a stable sample of outpatients as it may be important to distinguish between those individuals who lack insight in the presence of acute psychopathology versus those who have stabilized or remitted and are still lacking in insight.

Based on previous data, we hypothesised that (i) grey matter volume in the frontal, anterior cingulate and temporal lobe regions would be positively associated with one or more insight factor scores, and (ii) grey matter volume in the orbital/medial frontal region would be associated with the Awareness of and Attribution to Illness insight factor. As the specific dimensions of insight under investigation in our study were derived from a factor analysis, we expected them to associate primarily with grey matter volumes in different brain regions.

## 2 Methods and materials

### 2.1 Participants

This was a cross-sectional study and involved 52 outpatients with schizophrenia or schizoaffective disorder (diagnosed using the Structured Clinical Interview for DSM-IV Research Version; SCID-P, Spitzer et al., 1994). The patients included in this investigation were drawn from the larger sample of patients (*n* = 65) described in detail in a recent paper (Cooke et al., 2007). Thirty healthy participants recruited from the local community were also studied for comparison purposes. Exclusion criteria for healthy participants included a history of mental illness, drug and alcohol abuse or a regular medical prescription.

All patients were on stable doses of antipsychotic medication for at least 3 months prior to taking part in this study, and were in a stable (chronic) phase of the illness, living in the local community. The reasons for non-inclusion of 13/65 patients (reported in Cooke et al., 2007) were: unsuitable for magnetic resonance imaging (MRI; previous metalwork, claustrophobic or over-weight) (*n* = 7), consent withdrawal prior to MRI (*n* = 1), and movement artefacts /incomplete scan (*n* = 5). Excluded patients did not differ significantly from those included in the study in terms of age, predicted IQ, symptoms, or any of the insight variables (all *p* > 0.1).

Table 1 shows demographic characteristics of both study groups and Table 2 shows clinical characteristics of the patient sample.

**Table 1:** Demographic characteristics of study groups

		Patients (*n* = 52)	Controls (*n* = 30)	Statistics
		Mean (SD)	Mean (SD)	
		[Range]	[Range]	
Gender	(Male/Female)	40/12	24/6	*χ*^2^_1_ = 0.105
				*p* = 0.746
Age	(Years)	38.35 (9.89)	32.13 (12.38)	*T*_80_ = 2.495
		[19–61]	[20–65]	*p* = 0.015
Handedness	Edinburgh handedness	8.76 (2.27)	8.55 (1.31)	*T*_77_ = 0.472
	Inventory^a^	0.5–10.0	5.5–10.0	*p* = 0.638
Parental socioeconomic status		(*N* = 50)^b^	(*N* = 30)	Some cells too small for *χ*^2^
	Professional	7	8	
	Intermediate	25	10	
	Skilled: non-manual	3	7	
	Skilled: manual	12	3	
	Semi-skilled manual	1	1	
	Unskilled manual	2	1	
	Professional/intermediate	32	18	*χ*^2^_1_ = 0.13
	Other	18	12	*p* = 0.721

a Edinburgh handedness inventory (Oldfield, 1971).

b Reliable information not available for two patients.

**Table 2:** Clinical characteristics of patients (*n* = 52)

Patient characteristic		*n*	
Diagnosis	Schizophrenia	47	
	Schizoaffective disorder	5	
Medication	Atypical antipsychotic	42	
	Typical antipsychotic	10	

		Mean (SD)	Range
Duration of illness	Years	13.9 (9.6)	1–43
PANSS scores	Positive symptoms	16.5 (4.9)	7–28
	Negative symptoms	18.0 (4.9)	7–32
	General psychopathology	31.8 (6.3)	18–48
	Total	66.2 (13.7)	39–108

The study procedures had the approval of the ethics committee of the Institute of Psychiatry and South London and Maudsley Foundation NHS Trust, London. All participants provided written informed consent.

### 2.2 Measures of insight

Insight was assessed using a self-rated instrument, the Birchwood Insight Scale (Birchwood et al., 1994), and an observer-rated instrument, the Schedule for the Assessment of Insight — Expanded (Kemp and David, 1997). The SAI-E measures the dimensions of re-labelling of unusual mental events as abnormal, awareness of illness, and recognition of the need for treatment included in the original SAI (David et al., 1992). The SAI-E adds items regarding awareness of psychological/emotional changes, awareness that there is something wrong, awareness of the negative effects of mental illness, and attribution of symptoms to a mental illness. Like the SAI-E, the BIS is based on David's (1990) model of insight and measures the same three core dimensions of insight as the original SAI (David et al., 1992).

These two measures were combined, via a factor analytic approach, to generate four factors of insight, namely, Awareness of and Attribution to Illness, Recognition of the Need for Medication, Awareness of Problems and Symptom Re-labelling as described in detail by Cooke et al. (2007). Briefly stated, the Awareness of and Attribution to Illness factor includes items relating to awareness of illness, together with the attribution of problems in general as well as symptoms specifically to a mental illness. The Recognition of the Need for Medication factor includes items relating to awareness of the need for treatment, specifically medication. The Awareness of Problems factor includes items related to awareness of problems and the need to seek help. More specifically, the items which refer to being aware that one needs to take medication load on Recognition of the Need for Medication, whereas the items which refer to the awareness of the need to seek help more generally, and being aware that there was ‘something wrong’ load on Awareness of Problems factor. The Symptom Re-labelling factor comprises items referring to the ability to recognise that experiences are abnormal and label them as such but not perceive them as a problem or an illness. An individual may be aware that his/her symptomatic experiences are unusual or internally generated, but not attribute them to a mental illness and therefore score highly on the Symptom Re-labelling factor but not on Awareness of and Attribution to Illness.

### 2.3 MRI data acquisition

Structural MRI brain scans were acquired using a 1.5 Tesla GE N/Vi Signa system (General Electric, Milwaukee WI, USA) at the Maudsley Hospital, London. A quadrature birdcage head coil was used for RF transmission and reception. Head movement was limited by foam padding within the head coil and a restraining band across the forehead. Initially, a series of sagittal and axial fast gradient echo scout images were acquired in order to correct for head tilt and to orient subsequent images relative to the anterior-commissure/posterior-commissure line and the interhemispheric fissure (TR = 200 ms, TE = 4.2 ms, theta = 90°, field of view = 24 cm, slice thickness = 5 mm, slice gap = 2.5 mm, 256 × 192 acquisition matrix, one data average). The whole brain was then scanned with a 3-D inversion recovery prepared fast spoiled GRASS T1-weighted dataset. These T1-weighted images were obtained in the axial plane with 1.5 mm contiguous sections. TR was 18 ms, TI was 450 ms, TE was 5.1 ms and the flip angle was 20° with one data average, a 256 × 256 × 128 pixel matrix and a 0.937 mm × 0.937 mm in-plane resolution. Image contrast for all datasets was chosen with the aid of a software tool for optimizing image contrast (Simmons et al., 1996).

### 2.4 Optimised voxel-based morphometry procedure

All structural brain data were analysed using Statistical Parametric Mapping — version 2 (SPM2, Wellcome Department of Imaging Neuroscience, London; http://www.fil.ion.ucl.ac.uk/spm).

A series of study-specific, customised templates were created in preference to the standard SPM2 templates in order to minimise any scanner-specific biases and to provide templates appropriate to characteristics of the sample (Good et al., 2001). For the creation of the customised whole brain (T1) template, each structural MRI image was registered to the standard SPM2 T1 template using a 12 parameter affine transformation. These normalised images were then smoothed with an 8 mm full width at half maximum (FWHM) isotropic Gaussian kernel, and averaged to create the customised T1 template with a resolution of 1 mm × 1 mm × 1 mm.

The (unsmoothed) normalised T1 images were then segmented into their grey matter, white matter and cerebrospinal fluid components using the standard SPM2 grey matter, white matter and cerebrospinal fluid probability maps. Each individual segmented image was then automatically cleaned to remove non-brain tissue and smoothed with an 8 mm FWHM isotropic Gaussian kernel. The smoothed images were then averaged to create customised grey matter, white matter and cerebrospinal fluid templates in standard stereotactic space (Good et al., 2001).

The original T1 brain images were normalised to the customised T1 template using a 12 parameter affine transformation and then segmented into grey matter, white matter and cerebrospinal fluid components using the customised grey matter, white matter and cerebrospinal fluid templates. After automatically cleaning these segmented images to remove non-brain tissue, each grey matter image was re-normalised to the study-specific grey matter template using (i) a 12 parameter affine transformation and (ii) a non-linear transformation comprising a combination of smooth spatial basis functions (Ashburner and Friston, 1999) to account for global non-linear shape differences (16 iterations, 25 mm cut-off, medium regularisation). The parameters used to normalise each segmented grey matter image are then applied to the corresponding native T1 image, to derive a whole brain image which is normalised to standardised stereotactic space. Using the parameters from the normalisation of the cleaned segmented grey matter image to the grey matter template prevents non-brain voxels (such as the skull, which is present in T1 images) from contributing to errors in spatial normalisation, ‘optimising’ the process. These normalised whole brain T1 images, ‘optimised’ through using the parameters derived from the normalisation of grey matter images to a customised template, were used in the next step. In effect, the preceding stages of the process were concatenated into the normalisation of the native T1 images to maximise registration accuracy.

The initial normalisation of the T1 images to the T1 template prior to segmentation can be influenced by non-brain voxels (as the cleaning of these voxels from brain images can only be done once the images have been segmented, which must itself be preceded by an initial normalisation). The optimally normalised T1 images were therefore re-segmented into grey matter, white matter and cerebrospinal fluid using the customised grey matter, white matter and cerebrospinal fluid templates. These images are then cleaned to remove non-brain tissue to derive the optimally normalised segmented images for each subject.

In order to account for the volumetric changes introduced by non-linear transformations, images were modulated by multiplying the ‘relative volume’ (the extent of distortion generated by non-linear transformations) by the intensity value at each voxel in the (unsmoothed) optimally normalised, cleaned and segmented grey matter image. Differences between images in modulated intensities therefore reflect regional differences in the absolute amount (volume) of grey matter (Good et al., 2001). The images were then smoothed with a 12 mm FWHM isotropic Gaussian kernel, as recommended by Ashburner and Friston (2000) to ensure that the data are valid for the use of parametric statistical tests. The voxel size in these images was 1 mm × 1 mm × 1 mm.

### 2.5 Data analysis

The total grey matter volume for each individual was calculated from the unsmoothed modulated segmented images (which have the same grey matter volume as the segmented images in native space) and examined for (a) difference between patients and controls in total grey matter volume using analysis of variance with and without controlling for age and gender, and (b) the correlations between total grey matter volumes and insight measures (Pearson's *r* used for insight variables distributed normally, Spearman rank order correlations used when insight measures not normally distributed). Following the observation of positive associations between the total grey matter volume and the Awareness of Problems and Symptom Re-labelling factors of insight, we examined the relationships between (a) predicted IQ (assessed with National Adult Reading Test, NART; Nelson and Willison, 1991) and total grey matter volume, and (b) predicted IQ and insight factors. Subsequent to the observations of significant positive associations between predicted IQ and the total grey volume, and between predicted IQ and the Awareness of Problems and Symptom Re-labelling factors of insight, we re-evaluated the relationships between the total grey matter volume and relevant insight factors (Awareness of Problems and Symptom Re-labelling) after controlling for predicted IQ.

Regional differences between patients and controls in grey matter volume were examined by analysing group differences in modulated grey matter images. Both age and gender were controlled for in these analyses due to their known influence on brain structure (Coffey et al., 1998). To examine insight-regional grey matter associations in patients, a separate regression analysis was carried out for each insight factor. The effect of global grey matter volume was controlled for in the regression, and insight factor score was entered as an independent variable. Each within-group analysis of modulated grey matter images comprised two contrasts: one to test for positive associations between regional absolute grey matter volumes and insight measures, and another to test for negative associations. In order to exclude voxels which are unlikely to be grey matter from the analyses and reduce the number of comparisons being made, only voxels which had an absolute intensity of at least 0.05 (5%) in all grey matter images being analysed (i.e. smoothed, modulated normalised images) were included in each analysis.

The threshold for statistical parametric maps in this study was set at *p* < 0.001, uncorrected (using the *T* statistic in voxel-wise analyses). The threshold for local maxima to be considered significant was set at *p* < 0.05, family-wise error (FWE) corrected. The procedure for correction for multiple comparisons in SPM2, however, was originally designed for the analysis of functional data, and is overly strict when applied to structural data (Sowell et al., 1999). Relationships between insight and brain regional volumes where clusters were larger than 100 voxels with a local maxima reaching *T* > 4.50 (*p* < 0.0001) were therefore treated as being of interest. If these clusters were in a region which was hypothesised in advance to be related to insight, small volume correction (SVC) was applied to determine whether a cluster is significant correcting for multiple comparisons within a locally defined volume rather than the whole brain. A 10 mm radius sphere centred on the maxima voxel was used in SVC analyses.

The values representing the percentage of total grey volume under a smoothing kernel for each patient at peak voxels of the regions that showed an association with insight factors (see Results) were extracted and examined first using Pearsons' correlations and then re-examined using partial correlations after partialling out the effect of duration of illness in the observed associations (within SPSS). All significant associations remained significant with very similar *p* values after we partialled out the effect of duration of illness (results not presented further) most likely because neither the Insight factors nor the percentages of total grey volume in relevant regions were correlated with the duration of illness in this sample (all *p* > 0.10).

Finally, we explored possible associations between insight factors and white matter and CSF volumes (using the same methods and thresholds for significance testing as described above for the grey matter volume). The global volumes of white matter and CSF did not correlate significantly with any of the insight factors. The analyses to explore associations between regional white matter and each of the insight factor scores also did not reveal any significant relationships (all *T* < 4.5; results not presented further).

## 3 Results

### 3.1 Patients versus controls

#### 3.1.1 Global grey matter volume

Patients had significantly smaller global grey matter volume (in litres) than healthy controls (patients' mean = 0.716, SD = 0.066; controls' mean = 0.762, SD = 0.070; *F* = 9.299, *df* = 1,80, *p* = 0.003). This difference remained significant after controlling for age and gender (*F* = 6.607, *df* = 1,78, *p* = 0.012).

#### 3.1.2 Regional grey matter volume

Patients had significantly less grey matter volume in two regions: the first had its local maximum voxel in the right brainstem [(*x* = 9, *y* = −28, *z* = ,−8), *T*(78) = 5.54, number of contiguous voxels = 8540, *p* = 0.004 FWE corrected] and extended into the dorsal thalamus, and the second was located in the left middle occipital gyrus [BA 19, centred at − 53,− 82,− 1, *T*(78) = 4.80, number of contiguous voxels = 24468, *p* = 0.012 FWE corrected]. There was a trend towards less grey matter volume in the right superior temporal gyrus [STG, BA 38, centred at 55,16,− 8, *T*(78) = 4.34, number of contiguous voxels = 17,680, *p* = 0.079 FWE corrected].

There were no regions showing more grey matter volume in patients relative to controls (no voxels reached significance at the *p* < 0.001 uncorrected level).

### 3.2 Relationships between insight and GM volume

The mean scores for the four insight factors are described in Table 3. The associations reported between insight factors and MRI values are all positive — i.e. between greater grey matter volume and higher insight scores (see Table 4).

**Table 3:** Descriptive statistics of insight factors

	Insight factors			
	AAI^a^	RNM	AP	SR^a^
*n*	45	51	52	45
Mean	9.86	4.78	4.31	3.39
SD	4.98	1.78	2.02	1.93
Minimum	0	0	0	0
Maximum	16	6	6	6

Higher scores in each measure reflect greater insight. The maximum scores are the maximum scores possible for each factor. AAI — Awareness of and Attribution to Illness. RNM — Recognition of the Need for Medication. AP — Awareness of Problems. SR — Symptom Re-labelling.

a 7 patients did not possess any marked symptoms for which insight could be rated.

**Table 4:** Relationships between grey matter volume and insight factors

Brain region	BA	Cluster size^⁎^ (voxels)	Maxima voxels			*T* value	*p* value uncorrected	*p* value FWE corrected	*p* value SVC^ corrected
			*x*	*y*	*z*				
*Awareness of and Attribution to Illness*									
Left STG	21	2214	− 67	1	6	4.83	< 0.001	0.115	< 0.001
Left MTG	21	1487	− 73	− 42	− 4	4.52	< 0.001	0.242	0.001
Right ITG	21	709	71	− 5	− 23	4.68	< 0.001	0.166	0.001
Right inferior parietal lobule	40	6445	72	− 38	32	4.63	< 0.001	0.187	0.001
Right supramarginal gyrus			63	− 58	34	4.50	< 0.001	0.251	0.001

*Awareness of Problems*									
Left precuneus	7	1581	− 2	− 65	66	5.01	< 0.001	0.049	

*Symptom Re-labelling*									
Right STG	21	3775	66	− 7	− 8	4.56	< 0.001	0.224	0.001

BA — Brodmann area. FWE — Family-Wise Error. ^⁎^Cluster size at the uncorrected threshold of *p* = 0.001. ^Small Volume Correction — FWE corrected for 10 mm radius sphere search volume. STG — Superior temporal gyrus. MTG — Middle temporal gyrus. ITG — Inferior temporal gyrus.

#### 3.2.1 Global grey matter volume

Greater global grey matter volume was significantly associated with higher Awareness of Problems (rho = 0.275, *p* < 0.05) and higher Symptom Re-labelling scores (*r* = 0.315, *p* < 0.05). The Awareness of and Attribution to Illness and Recognition of the Need for Medication factors also showed positive associations with grey matter volume, but were not significant (*r* = 0.151 and *r* = 0.186 respectively, all *p* > 0.1).

Predicted IQ correlated positively with total grey matter volume (*r* = 0.385, *p* = 0.006, *n* = 50) and also with the Symptom Re-labelling (*r* = 0.328, *p* = 0.030) and Awareness of Problems scores (rho = 0.330, *p* = 0.019) but did not correlate significantly (*p* > 0.1) with the Awareness of and Attribution to Illness, and Recognition of the Need for Medication scores. The association between the total grey matter volume and relevant insight factors (Awareness of Problems and Symptom Re-labelling) became non-significant (*p* > 0.1) after controlling for predicted IQ.

#### 3.2.2 Regional grey matter volume

##### 3.2.2.1 Awareness of and Attribution to Illness

**Fig. 1:**
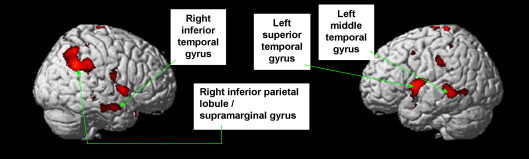
Association between Awareness of and Attribution to Illness score and absolute volume of the left superior temporal gyrus, right inferior temporal gyrus, and right inferior parietal lobule/supramarginal gyrus.

There was an association between higher score on Awareness of and Attribution to Illness and greater grey matter volume in the left STG, as well as a more posterior region in the left middle temporal gyrus. In the right hemisphere, this factor was associated with the volume of the right inferior temporal gyrus and a large lateral parietal cluster with two local maxima: one in the right inferior parietal lobule and another in the right supramarginal gyrus (see Table 1 and Fig. 1).

##### 3.2.2.2 Recognition of the Need for Medication

There were no significant correlations between this factor score and regional grey matter volumes (all voxels *T* < 4.5 and *p* > 0.3, FWE corrected).

##### 3.2.2.3 Awareness of Problems

**Fig. 2:**
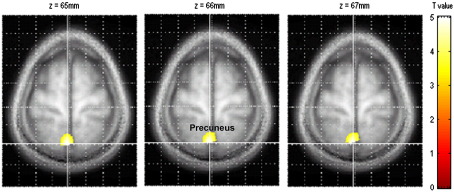
Association between greater absolute grey matter volume in the left precuneus and the Awareness of Problems insight factor.

There was a significant association between higher score and greater grey matter volume in the left precuneus (see Table 3 and Fig. 2).

##### 3.2.2.4 Symptom Re-labelling

**Fig. 3:**
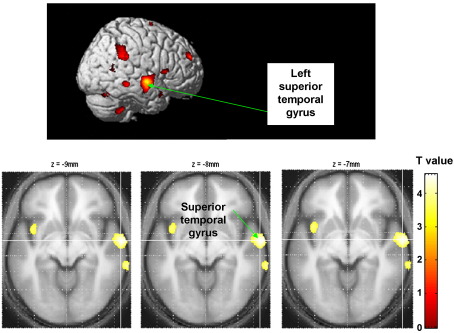
Association between greater absolute grey matter volume in the right temporal gyrus (the two other clusters seen were not significantly associated) and the Symptom Re-labelling insight factor.

There was an association between higher score and greater absolute grey matter volume in the right STG (see Table 3 and Fig. 3).

## 4 Discussion

A number of studies have suggested that insight in schizophrenia has a structural brain basis. Frontal brain regions have been particularly implicated, although ROI studies have rarely examined regions outside of the frontal lobes. This investigation aimed to extend the literature by using VBM, an automated approach which covers the whole brain, to examine the structural brain correlates of clinical insight and its specific dimensions.

### 4.1 Patients versus controls

The patient group had a significantly smaller total grey matter volume compared to controls, a difference which survived controlling for age and sex. This result is consistent with the global reduction in grey matter volume in individuals with schizophrenia found in a meta-analysis (Wright et al., 2000). Regionally, there were significant grey matter reductions in patients in a right brainstem/dorsal thalamus cluster and the left middle occipital gyrus (BA 19), as well as a trend towards a reduction in the right STG (BA 38). These findings are consistent with those of a number of previous VBM studies. Four studies have found reductions in grey matter in the right thalamus in patients with schizophrenia compared to controls (Hulshoff Pol et al., 2001, Marcelis et al., 2003, Salgado-Pineda et al., 2003, Salgado-Pineda et al., 2004), while left middle occipital gyrus reductions have been found in one previous study (Moorhead et al., 2004). Reductions in the right STG are one of the most replicated VBM findings (Sigmundsson et al., 2000, Hulshoff Pol et al., 2001, Job et al., 2002, Kubicki et al., 2002, Spalletta et al., 2003, Moorhead et al., 2004). The STG finding is also in accord with findings of ROI studies (Shenton et al., 2001). Reductions in the right STG in schizophrenia are thought to be particularly important in the aetiology of auditory hallucinations, as STG volume has been found to correlate with the severity of such experiences (Barta et al., 1990). However, it should be noted that, although no healthy participant or patient was abusing drugs or alcohol at the time of their participation, there was a past history of drug abuse in a proportion of patients and this may have led or contributed to some of the observed grey matter differences between patients and controls.

### 4.2 Insight and grey matter volume

It was hypothesised that insight factors would be correlated with total grey matter volume. This hypothesis was supported by the significant correlations between total grey matter volume and both the Awareness of Problems and Symptom Re-labelling factors. However, predicted IQ was also correlated with the total grey matter volume and the Awareness of Problems and Symptom Re-labelling factors and the associations between total grey matter volume and the Awareness of Problems and Symptom Re-labelling factors became non-significant after controlling for predicted IQ. Generally, our findings are consistent with previous data showing (a) a relationship between IQ and total grey matter volume (review, Antonova et al., 2004) and (b) a relationship between insight and general cognitive ability in psychosis (reviews, Cooke et al., 2005, Aleman et al., 2006). Our findings, however, also suggest that the total brain volume has a noticeable association only with Symptom Re-labelling and Awareness of Problems dimensions of insight and this effect is mediated via its association with general cognitive ability.

A number of previous (mainly ROI) studies have found relationships between dimensions of insight and the volume of specific frontal lobe regions. However, no significant relationships were found between the insight factors and frontal lobe regions in this study. Instead, we found associations between insight factors and a number of temporal and parietal lobe areas. Previous ROI studies have not measured parietal or temporal lobe regions, making it impossible for them to identify correlations between the volume of these regions and insight. Furthermore, anosognosia, which is held by some to be the neurological analogue of poor insight (Amador et al., 1994), is associated with lesions to subcortical, parietal lobe and temporal lobe structures, as well as with frontal lesions (meta-analytic review, Pia et al., 2004), suggesting that it is not a purely frontal phenomenon.

The strongest relationship found in this investigation was between the Awareness of Problems score and the volume of the left precuneus, located in the medial parietal lobe. This relationship survived FWE correction for multiple comparisons without the need for small volume correction. A number of studies have demonstrated that grey matter volumes of specific brain regions are closely associated with behavioural measures of their function both in healthy people and in patients with schizophrenia (review, Antonova et al., 2004). Although the precuneus has not been investigated in previous structural imaging (ROI) studies within the context of schizophrenia (as discussed by Antonova et al., 2004), functional imaging studies in healthy people have revealed a number of observations concerning its functions that allow us to postulate how it might play a role in this particular dimension of insight in psychosis. Specifically, a network involving the precuneus and medial prefrontal regions has been proposed as the mechanism through which personal identity and past personal experiences are interlinked, allowing a person to move between representation and awareness of the self (Andreasen et al., 1995). Functional magnetic resonance imaging studies have shown precuneus activation in comparing self to non-self representations, including judging whether psychological/personality trait adjectives were self-referential (Kircher et al., 2000, Kircher et al., 2002). Positron emission tomography studies too have shown precuneus activation when participants reflect about their own personality traits and physical appearance (Kjaer et al., 2002), and a linear relationship between precuneus activation and the degree to which the retrieval of previous retrieval of psychological traits was self-referential (Lou et al., 2004). These observations indicate that the precuneus is either involved in assigning first-person perspective (e.g. awareness of one's own mental states) (Vogeley and Fink, 2003), or more generally in internal representation through mental imagery and episodic/autobiographical memory retrieval (Cavanna and Trimble, 2006, Cavanna, 2007) and could explain why the volume of the left precuneus was associated with the Awareness of Problems. If an individual with schizophrenia has a deficit in an area of the brain which mediates the ability to access representations of his/her own mental states, he/she may not be able to identify that these mental states are problematic, and may therefore be unaware that there is ‘something wrong’. We (Cooke et al., 2007) had previously found an association between lower awareness of problems and ‘preference for mental disengagement’ coping style. This raises an interesting possibility that precuneus deficit causes mental disengagement via reducing awareess of one's own mental states.

A large cluster in a different parietal lobe region was correlated with Awareness of and Attribution to Illness factor score. This cluster had maxima voxels in the right inferior parietal lobule and right supramarginal gyrus, and was significant after small volume correction had been applied. This area of the parietal cortex has a well-established role in visual attention (review, Culham and Kanwisher, 2001) and working memory/executive functioning tasks that also activate the DLFPC (Bor and Owen, 2007). It is plausible that as well as being involved in awareness of sensory information, the right inferior parietal cortex is involved in awareness of illness via the same cognitive dysfunctions, namely deficits in executive working memory and cognitive set shifting, that link the DLPFC deficit to poor insight in patients with schizophrenia (review, Shad et al., 2007). In our recent study (Cooke et al., 2007), we had found ‘Awareness of and Attribution to Illness’ factor score to be correlated with ‘planning’ and ‘use of instrumental social support’ styles of coping which may also rely on the same set of cognitive functions. It is also interesting to note that damage to the right parietal cortex has frequently been linked to anosognosia in neurological disorders (Pia et al., 2004, Vuilleumier, 2004).

The present investigation found relationships between the volume of a number of temporal lobe regions and the Awareness of and Attribution to Illness insight factor. Ha et al. (2004) had previously reported an association between insight, as measured by the PANSS G12 item, and a temporal lobe grey matter cluster. Our findings suggest a role for grey matter in the temporal lobe specifically in the awareness of symptoms and attributing them to an illness rather than in insight across all dimensions. In the present investigation, two clusters in the left temporal lobe were significant after small volume correction had been applied: one with a maxima voxel in the left STG, and another more posterior cluster with a maxima voxel in the left middle temporal gyrus. A further cluster in the right inferior temporal gyrus was also significant after applying small volume correction. A cluster with its maxima voxel in the right middle temporal gyrus which extended into the right STG was also associated with the Symptom Re-labelling factor and was significant after small volume correction had been applied. Functional neuroimaging studies have demonstrated the role of the STG in the cortical network subserving the interpretation, production and self-monitoring of speech and language in healthy people (review, Pearlson, 1997). Activation of this region has been found to correlate with severity of auditory hallucinations in people with schizophrenia (Barta et al., 1990; review, Raveendran and Kumari, 2007), supporting the view that auditory hallucinations reflect impaired self-monitoring of internal speech (Frith, 1992). The correlations found in this study suggest that the self-monitoring deficits in schizophrenia, which associated with the STG, may extend beyond an inability to monitor internal speech, to an inability to monitor whether mental experiences are abnormal, and whether one is experiencing a psychiatric illness.

Shad et al. (2007) have very recently proposed that reduced volumes of the right DLPFC and/or the right parietal regions underlie unawareness of symptoms (anosognosia), while relatively increased volumes of the orbitofrontal cortex underlies misattribution of symptoms (dysnosgnoia). Our data provide support for the theorised parietal cortex–insight relationship. The lack of any inverse relationship between any dimensions of insight and brain volumes especially of the orbitofrontal region may be due to the fact that all our patients were medicated and chronic outpatients. Very recently, Lacerda et al. (2007) using the same ROI procedures as used in studies by Shad and colleagues have reported larger orbitofrontal volume in first-episode schizophrenia patients. Patients diagnosed with schizophrenia who have long term difficulties on the other hand are reported to show reduced grey matter volume in this region (Kuperberg et al., 2003). It is thus possible that a misattribution of symptoms–larger orbitofrontal volumes relationship is specific to first-episode patients. However, unlike the findings of ROI studies, grey matter volume in no frontal region associated positively or negatively with insight dimensions in this study. This, at least in part, may be related to the methodological differences between voxel-averaged, landmark-based ROI analyses and the single, voxel-by-voxel whole brain VBM measurements (Gilulani et al., 2005).

We did not find any association between the ‘Recognition of the Need for Medication’ factor and total or regional grey matter volumes. Although we earlier suggested that this insight dimension may be less biological in nature and better correlated with psychological mechanisms, such as denial, poor self-esteem or a coping style (Sapara et al., 2007), our recent study did not show an association between this dimension and denial, poor self-esteem or a particular coping style (Cooke et al., 2007). The biological, psychological and/or social variables that influence, or may be associated with, this particular dimension of insight remain unclear at present.

## 5 Conclusions

The findings of this study demonstrate positive relationships between total grey matter volume and grey matter volumes in a number of regions and specific dimensions of insight in psychosis. Specifically, greater left precuneus grey matter volume, perhaps via facilitating increased access to own mental states, has a positive association with awareness of problems and the need to seek help. Greater grey matter volumes in the STG, a region implicated in self-monitoring and perhaps in an extended capacity in the ability to monitor whether mental experiences are abnormal and whether one is experiencing a psychiatric illness, and the lateral parietal cortex, which has a role in working memory/executive functioning, are associated with higher awareness of symptoms and attributing them to an illness.

## Role of the funding source

The sponsor (the Wellcome Trust) had no role in the study design; in the collection, analysis and interpretation of data; in the writing of the report; or in the decision to submit the paper for publication.

## Contributors

Veena Kumari, Elizabeth Kuipers, Emmanuelle Peters, Steven C Williams and Michael Cooke designed the study and obtained the funding. Michael Cooke collected the data. Dominic Fannon performed the clinical diagnostic interviews. Michael Cooke undertook the statistical analysis. Veena Kumari and Michael Cooke prepared the first draft. All authors contributed to and approved the final manuscript.

## Conflict of interest

The authors declare no conflict of interest.
